# Transtheoretical Model (TTM)-Based, TTM-Informed and TTM-Congruent Behaviour Change Interventions for Adults with Mild Cognitive Impairment and Dementia Risk: A Scoping Review

**DOI:** 10.3390/healthcare14131898

**Published:** 2026-06-30

**Authors:** Wei Ting Foo, Xiaoting Huang, Shawn Zhi Zheng Lin, Anupama Roy Chowdhury

**Affiliations:** 1 Department of Geriatric Medicine, Singapore General Hospital, Singapore 169608, Singapore; huang.xiaoting@singhealth.com.sg (X.H.); anupama.roy.chowdhury@singhealth.com.sg (A.R.C.); 2 Department of Neurology, National Neuroscience Institute, Singapore General Hospital Campus, Singapore 168581, Singapore; shawn.lin.z.z@singhealth.com.sg

**Keywords:** mild cognitive impairment, dementia prevention, transtheoretical model, behaviour change interventions

## Abstract

**Highlights:**

**What are the main findings?**

- Despite growing interest in behaviour change for dementia prevention, explicit TTM-based interventions for adults with MCI remain scarce, with most published studies using TTM-informed or TTM-congruent behavioural approaches instead.
- The strongest and most consistent signals were seen in behavioural and intermediate outcomes—such as adherence, self-management, dietary change, and sustained physical activity—whereas cognitive benefits were less consistent and less durable.

**What are the implications of the main findings?**

- Current evidence does not establish TTM-specific cognitive efficacy in MCI. The TTM is best understood as a readiness and maintenance lens for intervention design in this field rather than as a proven mechanism for cognitive improvement.
- Future studies should measure TTM constructs directly, distinguish engagement from actual behaviour change, and test whether stage-matched approaches improve behavioural, cognitive, or functional outcomes beyond non-stage-matched behavioural support.

**Abstract:**

**Background/Objectives:** Mild cognitive impairment (MCI) is a clinically important state associated with an increased risk of future cognitive decline and dementia. Behaviour change interventions may support risk reduction and self-management in cognitively vulnerable adults. However, the extent to which the transtheoretical model (TTM) has been used in this population has not been clearly mapped. This scoping review examined TTM-based, TTM-informed, and TTM-congruent behaviour change interventions for adults with MCI, subjective cognitive concerns, or elevated dementia risk. **Methods:** This scoping review followed Joanna Briggs Institute guidance and was reported in accordance with PRISMA-ScR. The protocol was prospectively registered on the Open Science Framework. PubMed, PsycINFO, ScienceDirect, Scopus, Web of Science, CENTRAL, ProQuest Dissertations, and medRxiv were searched from inception to 30 September 2025. Eligible studies included randomised, nonrandomised, quasi-experimental, and qualitative designs. Data were charted using a piloted extraction form and were synthesised narratively. **Results:** Eight unique studies, represented across nine publications, were included. Only one trial explicitly operationalised the TTM in a clinically defined MCI cohort; most studies were more appropriately classified as TTM-informed or TTM-congruent. Recurrent intervention components included readiness alignment, goal setting, self-monitoring, personalised feedback, prompts and cues, problem solving, reinforcement, and relational support. Behavioural outcomes were more consistently favourable than cognitive outcomes, particularly for adherence, self-management, diet, and sustained physical activity engagement. Cognitive findings were heterogeneous: some smaller studies reported short-term improvements, whereas the largest rigorous trial found no significant cognitive benefit. **Conclusions:** Current evidence does not support strong claims regarding TTM-specific cognitive efficacy in MCI. Instead, it suggests that TTM-informed and TTM-congruent interventions may be useful for strengthening behavioural regulation, risk reduction, and maintenance of health-related routines in cognitively vulnerable adults. More rigorous studies are needed to test the TTM constructs prospectively and to determine whether proximal behavioural change translates into durable cognitive or functional benefit.

## 1. Introduction

Mild cognitive impairment (MCI) is a clinically important state because it lies between normal cognitive ageing and dementia and is associated with a substantially increased risk of future decline. Although the clinical trajectory of mild cognitive impairment (MCI) is heterogeneous, with some individuals remaining stable and others showing transient reversion to normal cognition, a diagnosis of MCI remains clinically meaningful because it predicts future outcomes. Even individuals who appear to revert to normal cognition continue to have a higher risk [1,2] of later cognitive impairment or dementia than those who never had MCI in the first place.

This combination of vulnerability and instability makes MCI a clinically relevant target for interventions that aim to preserve cognition, strengthen self-management, and reduce longer-term risk. Recent epidemiologic studies estimated a pooled prevalence of 15.56 percent for MCI among community-dwelling adults aged 50 years and older [3]. Recent regional evidence indicates that cognitive impairment and dementia prevalence across Asia [4] are substantial and rising, with important geographical variations and uneven surveillance. Longitudinal evidence [5] also indicates that MCI trajectories are dynamic, with progression, persistence, and reversion all observed over time.

Over the past decade, dementia prevention research has increasingly focused on modifiable risk factors [6] across the life course. Physical inactivity, poor diet, hypertension, diabetes, smoking, depression, social isolation, and other potentially alterable exposures are now central to risk-reduction strategies. This shift has led to growing interest in multidomain interventions [7] that combine behavioural support with vascular and lifestyle risk management. Major guidance documents and prevention frameworks now treat risk reduction as a core part of dementia prevention rather than a peripheral adjunct. This broader prevention agenda is supported by intervention evidence showing that multidomain lifestyle programmes can improve cognitive outcomes in at-risk older adults. In the Finnish Geriatric Intervention Study to Prevent Cognitive Impairment and Disability [8], a two-year multidomain intervention that combined diet, exercise, cognitive training, and vascular risk monitoring led to better overall cognitive performance than controls, providing proof of principles that structured risk reduction can modify clinically relevant trajectories.

In this review, clinically defined MCI was treated as the anchor population. Subjective cognitive concerns, elevated dementia-risk cohorts, midlife Alzheimer disease risk cohorts, and care-partner dyads were treated as adjacent populations along a cognitive-risk and prevention continuum. These adjacent populations were included because behaviour change interventions for dementia prevention are often implemented before, around, or shortly after the emergence of measurable cognitive impairment. Findings from these groups were not interpreted as directly equivalent to findings from clinically defined MCI.

The translational challenge is that dementia-risk reduction recommendations do not, by themselves, produce sustained behaviour change. This challenge may be greater among older adults with cognitive vulnerability, in whom memory impairment, reduced confidence, multimorbidity, treatment burden, and reliance on family or care partners may affect the initiation and maintenance of health-related routines. The transtheoretical model (TTM) [9] is an appropriate focal model for this review because it was developed as an integrative model of intentional behaviour change and explicitly links stages of change with processes of change, decisional balance, self-efficacy, and temptation. These constructs correspond closely to common clinical barriers in MCI and dementia-risk reduction: low readiness, ambivalence, difficulty sustaining routines, variable confidence, relapse after initial improvement, and the need for maintenance support.

The choice of TTM does not imply that it is the only relevant behaviour change model. Its value for this review is its specific focus on readiness and maintenance, which makes it a useful lens for examining whether interventions are tailored to a person’s position in the change process [9]. This is particularly relevant in cognitively vulnerable populations, where health education, risk-reduction advice, and lifestyle prescriptions may fail if they assume that individuals are already ready, confident, and able to sustain change. A scoping review is therefore appropriate because it can determine whether the TTM has been explicitly operationalised, whether its constructs have been measured, and whether adjacent interventions merely use TTM-congruent components without testing the model.

The model should nevertheless be interpreted cautiously. The TTM has been criticised for assuming relatively discrete stages of change, for limited evidence that stage-matched interventions consistently outperform non-stage-matched interventions, and for the tendency to classify common behaviour change techniques as TTM-specific when they are also used in other frameworks [10,11,12]. These concerns are particularly relevant in MCI and dementia-risk populations, where behaviour change may be nonlinear, cognitively constrained, caregiver-mediated, and shaped by environmental and health-system factors.

This scoping review, therefore, did not seek to estimate the pooled efficacy of the TTM. Instead, it aimed to map how the TTM has been used in interventions for adults with clinically defined MCI and adjacent dementia-risk populations; to distinguish explicit TTM-based interventions from TTM-informed and TTM-congruent interventions; to describe intervention architecture and theoretical fidelity; and to summarise behavioural and cognitive outcome patterns.

## 2. Materials and Methods

### 2.1. Design and Registration

We conducted a scoping review following Joanna Briggs Institute methodological guidance and reported the review in compliance with the PRISMA extension for scoping reviews (PRISMA-ScR) [13,14]. The protocol was prospectively registered on the Open Science Framework (OSF). Because the relevant evidence spans randomised trials, nonrandomised studies, secondary analyses, and qualitative process work, the review was designed to map evidence structures, intervention architecture, theoretical fidelity, and outcome patterns rather than to estimate pooled comparative efficacy. This approach is consistent with contemporary methodological work that positions mapping reviews within the broader scoping review family when the aim is to clarify how an evidence base is organised and where evidence gaps remain.

### 2.2. Eligibility Criteria

This review used a population, concept, and context framework. The population of interest was adults with clinically defined MCI. Studies involving subjective cognitive concerns, elevated dementia risk, midlife Alzheimer disease risk, or care-partner dyads involving persons with MCI or mild dementia were eligible when the intervention targeted behaviour change that was relevant to cognitive health, dementia-risk reduction, self-management, or maintenance of health-related routines. Clinically defined MCI was treated as the anchor population during synthesis. Adjacent dementia-risk populations were analysed separately and used to map wider intervention architecture rather than to infer direct TTM efficacy in MCI.

The concept of interest was a TTM-based, TTM-informed, or TTM-congruent behaviour change intervention. A three-category classification was used to keep the review centred on the TTM while avoiding conflation between direct model testing and broader behavioural support.

Interventions were classified as TTM-based when the TTM was explicitly named as the primary intervention framework and when core TTM constructs were used to structure the intervention content, assessment, or tailoring. These constructs included the stage of change, stage-matched intervention content, stage progression, decisional balance, self-efficacy, processes of change, temptation, relapse prevention, or maintenance.

Interventions were classified as TTM-informed when the TTM was explicitly referenced or when identifiable TTM constructs were used, but the model was not fully operationalised as the primary intervention framework.

Interventions were classified as TTM-congruent when they did not explicitly test the TTM but incorporated behaviour change components that overlap with TTM constructs or processes, including goal setting, self-monitoring, prompts and cues, personalised feedback, problem solving, reinforcement, graded practice, social support, and maintenance support. TTM-congruent studies were not treated as evidence of TTM efficacy. They were included because preliminary searching showed that explicit TTM-based trials in clinically defined MCI were scarce, whereas TTM-compatible behaviour change components were common in interventions targeting dementia-risk reduction. These studies were used to define the boundary between explicit TTM testing and adjacent behaviour change architecture.

The context included clinical and community settings, including primary care, memory services, home-based programmes, and technology-enabled interventions. Randomised, nonrandomised, quasi-experimental, secondary analysis, and qualitative studies were eligible if they reported behavioural, cognitive, functional, intermediate health, or implementation-relevant outcomes. Opinion pieces, purely theoretical papers, protocols without outcome data, and interventions targeting only caregivers without a clear behavioural target for the person living with cognitive impairment were excluded. Non-English publications were also excluded.

### 2.3. Information Sources and Search Strategy

Searches were conducted in PubMed, PsycINFO, ScienceDirect, Scopus, Web of Science Core Collection, and Cochrane CENTRAL from inception to 30 September 2025. Additional searches included ProQuest dissertations and medRxiv, with backward and forward citation tracking of included studies and relevant reviews. Search terms combined free-text and controlled vocabulary terms for mild cognitive impairment, dementia risk, the transtheoretical model, stages of change, behaviour change, intervention exposure, and relevant behavioural, cognitive, functional, psychological, and quality-of-life outcomes. The full reproducible PubMed search strategy, including field tags, Boolean operators, and applied filters, is provided in Supplementary Index S1.

### 2.4. Study Selection

Searches of electronic databases and supplementary sources identified 2058 records. After the removal of 78 duplicates, 1980 records underwent title and abstract screening, of which 1832 were excluded. The remaining 148 full-text reports were assessed for eligibility. Full-text reports were excluded if they did not meet the prespecified population, concept, context, intervention, outcome, or publication type criteria.

**Figure 1:**
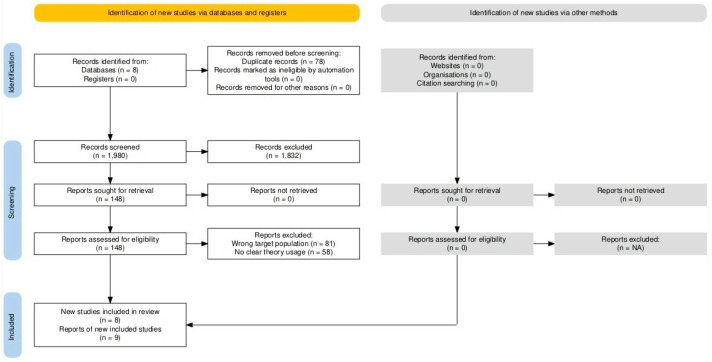
PRIMSA flow diagram.

Records were managed and screened using Rayyan (Rayyan Systems, Inc., Cambridge, MA, USA; https://www.rayyan.ai/) in two sequential stages: title and abstract screening, followed by a full-text review. Two reviewers independently screened records at each stage after an initial calibration exercise. Disagreements were resolved through discussion, with adjudication by a third reviewer where required. Reasons for exclusion were recorded at the full-text stage. The complete selection process is summarised in the PRISMA flow diagram below in Figure 1.

### 2.5. Data Charting

Data charting was guided by a pre-piloted extraction form developed in Microsoft Excel (Microsoft Corporation, Redmond, WA, USA; https://www.microsoft.com/en-us/microsoft-365/excel). For each included study, we charted bibliographic details, country and setting, study design, population, intervention and comparator content, duration, follow-up, outcomes, and main findings. We additionally charted the named theoretical framework, whether the TTM was explicitly cited, whether the stage of change was assessed, whether the intervention content was stage-matched, whether stage progression was measured, whether decisional balance, self-efficacy, temptation, or processes of change were assessed, and whether the intervention components reflected broader behaviour change techniques rather than TTM-specific constructs.

Theoretical fidelity was synthesised narratively across three levels: direct TTM operationalisation, partial or indirect TTM use, and TTM-congruent behaviour change architecture without direct TTM testing. Studies were not pooled conceptually across these levels. Findings from TTM-congruent studies were used to map intervention architecture and outcome patterns.

The extraction form was piloted independently by two reviewers on a subset of included reports and refined iteratively to improve clarity, consistency, and capture of theory-related variables. Following piloting, one reviewer completed full extraction, and a second reviewer checked all entries for accuracy and completeness against the source reports. Any discrepancies were resolved by consensus, with recourse given to a third reviewer if required. When multiple publications arose from the same study, data were charted at the publication level and then consolidated at the study level during synthesis in order to avoid double-counting while preserving distinct outcome reporting.

### 2.6. Critical Appraisal

Consistent with scoping review methodology, studies were not excluded on the basis of methodological quality, and no formal risk-of-bias grading was undertaken for quantitative comparison. We nevertheless conducted a structured methodological appraisal to support the interpretation of the evidence. For randomised and nonrandomised quantitative studies, we considered the allocation method, comparator condition, blinding where relevant, sample size, attrition, follow-up duration, outcome measurement, and the extent to which the design supported causal inference. For qualitative studies, we considered sampling, analytic transparency, and the relevance of the findings to feasibility, implementation, or behavioural mechanisms.

Appraisal findings were not converted into summary scores and were not used to determine eligibility. Instead, they informed the narrative synthesis by distinguishing evidence that could support efficacy-related interpretation from evidence that was more appropriately used to understand feasibility, implementation, intervention architecture, or plausible behavioural mechanisms.

### 2.7. Synthesis

Study characteristics were summarised descriptively, and findings were synthesised narratively. Meta-analysis was not undertaken because the evidence base was heterogeneous in population, intervention content, theoretical fidelity, study design, comparator condition, follow-up duration, and outcome measurements.

The primary analytic lens was theoretical operationalisation. Interventions were grouped as TTM-based, TTM-informed, or TTM-congruent. TTM-based interventions explicitly used the TTM as the principal framework. TTM-informed interventions referenced the TTM but operationalised it incompletely. TTM-congruent interventions incorporated behaviour change components aligned with TTM processes without formally presenting the intervention as TTM-based.

A second layer of synthesis examined intervention targets and outcomes. Behavioural outcomes were grouped into health management, physical activity, diet, oral hygiene, multidomain lifestyle risk reduction, and adherence or engagement. Cognitive outcomes were summarised separately because they were more distal, measured with different instruments, and less consistently reported. Functional, mood-related, cardiometabolic, and implementation-relevant outcomes were considered where reported.

Where multiple publications reported data from the same study, findings were integrated at the study level to avoid double-counting. In drawing interpretive conclusions, greater weight was placed on randomised designs, larger samples, longer follow-ups, and clearly defined comparators. Smaller, nonrandomised, secondary, and qualitative studies were used primarily to inform feasibility, implementation, intervention architecture, and behavioural plausibility.

## 3. Results

### 3.1. Overview of Included Studies

Eight unique studies met the inclusion criteria and were represented across the nine publications listed in Table 1 because the Gray Matters trial contributed both a primary randomised trial report [15] and a later secondary analysis of motivational outcomes [16]. The evidence base was organised around clinically defined MCI as the anchor population and adjacent dementia-risk populations as contextual evidence. Two studies focused on clinically defined MCI. Shi et al. [17] provided the only direct test of an explicitly TTM-based intervention in MCI, while Kim et al. [18] evaluated a self-care intervention in older adults with MCI. AIBL Active [19] included older adults with MCI or subjective memory complaints and cerebrovascular risk factors. MEDEX [20] enrolled older adults with subjective cognitive concerns but not dementia. AgeWell.de [21] and MedEx-UK [22] enrolled older adults at increased dementia risk. Gray Matters [15] enrolled midlife adults at elevated Alzheimer’s disease risk without significant cognitive impairment. Bryant et al. [23] examined care-partner dyads involving persons with MCI or mild dementia.

### 3.2. Critical Appraisal of Included Sources of Evidence

Critical appraisal was used to support interpretation rather than to determine eligibility. Overall methodological strength varied across the included evidence. Randomised controlled trials were given greater interpretive weight for efficacy-related outcomes, particularly where comparator groups, follow-up assessments, and prespecified outcomes were clearly reported. The explicit TTM-based randomised trial by Shi et al. [17] provided the most direct evidence in a clinically defined MCI population, although the follow-up duration limited the conclusions made about durability. Larger randomised trials such as AgeWell.de [21] and MEDEX [20] provided stronger methodological designs and longer follow-ups but were not explicit tests of the TTM and were conducted in broader dementia-risk populations. Nonrandomised, secondary, and qualitative studies were interpreted more cautiously and were used primarily to inform behavioural plausibility, intervention mechanisms, feasibility, and implementation. No study was excluded on the basis of methodological limitations.

Findings from this structured appraisal are summarised in Supplementary Index S2 and were used to guide interpretation rather than determine eligibility.

### 3.3. Theoretical Fidelity

Theoretical fidelity varied substantially across the included studies. Shi et al. [17] provided the only direct test of an explicitly TTM-based intervention in clinically defined MCI. The intervention used stage-aligned health education, weekly 45–60 min sessions over 8 weeks, followed by 12 weeks of unsupervised practice. The behavioural stage was measured using a five-stage cognitive-function health-management item, and disease knowledge and adherence to cognitive-function health-management behaviours were assessed at baseline, 8 weeks, and 20 weeks. This study, therefore, provided the strongest direct evidence for TTM operationalisation in this review.

Gray Matters [15] was classified as TTM-informed because the app design incorporated goal targets, self-monitoring, and reinforcement concepts, and the parent intervention explicitly included TTM-aligned reinforcement language. The latter motivational analysis [16] was more closely grounded in self-determination theory, assessing intrinsic motivation, amotivation, external regulation, and identified regulation. It was therefore retained as TTM-informed through the parent intervention but not treated as a direct TTM test.

All remaining studies were classified as TTM-congruent. Kim et al. [18] used Simmons’s Health-Promoting Self-Care System Model, informed by Orem’s Self-Care Deficit Nursing Theory, Pender’s Health Promotion Model, and Cox’s Interaction Model of Client Health Behaviour. It did not test the TTM stages or TTM mechanisms, but included components overlapping with self-efficacy, reinforcement, social support, and maintenance processes. AIBL Active [19], AgeWell.de [21], MEDEX [20], and MedEx-UK [22] used structured behavioural support, repeated contacts, personalised targets, web-based support, feedback, or maintenance phases, but did not measure TTM constructs. Bryant et al. [23] used behaviour change technique coding in care-partner coaching transcripts, identifying prompts and cues, instructions, goal reviews, and problem solving; these components overlap with TTM processes but were not operationalised as TTM constructs.

The review identified a narrow literature on explicit TTM use in MCI and a broader literature on TTM-congruent behaviour change architecture across adjacent dementia-risk populations. The latter supports conclusions about common intervention components, but not about TTM-specific efficacy.

### 3.4. Main Findings Across Studies

Across included studies, behavioural and implementation-related outcomes were more consistently favourable than cognitive outcomes. The explicit TTM-based RCT by Shi et al. [17] showed significant effects on behavioural stage, disease knowledge, and adherence at both 8 and 20 weeks, with global cognition improving significantly by 20 weeks. Kim et al. [18] reported significant improvements in dementia-preventive behaviour, self-efficacy, cognition, depression, and quality of life, but the quasi-experimental design and non-TTM theoretical basis limit causal and TTM-specific inference. AIBL Active [19] demonstrated high long-term adherence to home-based physical activity over 24 months, supporting the feasibility of sustained behavioural maintenance in selected older adults with cognitive concerns and cerebrovascular risk factors.

Digital and multidomain interventions showed domain-specific behavioural effects. Gray Matters [15] demonstrated high app engagement and associations between app use or goal attainment and selected cardiometabolic markers, while the latter motivational analysis showed increased intrinsic motivation but mixed subgroup associations with physical activity and diet quality. AgeWell.de [21] reduced LIBRA dementia-risk scores, mainly through diet and hypertension. MedEx-UK [22] improved Mediterranean diet adherence at 24 and 48 weeks, but objectively measured physical activity did not significantly change.

Cognitive findings were less consistent. Shi et al. [17] and MedEx-UK [22] reported short-term cognitive improvements, but MEDEX [20], the largest included study, found no significant improvement in episodic memory or executive function at 6 months or 18 months after mindfulness training, exercise, or their combination. This finding substantially limits claims that structured behavioural interventions in cognitively at-risk populations reliably translate into cognitive benefits. The behavioural, cognitive and related findings are found in Table 2.

## 4. Discussion

This review maps a small and uneven evidence base rather than an efficacy literature for TTM in MCI. Four findings address the review objectives. First, the evidence structure is sparse: only one included study directly tested an explicitly TTM-based intervention in clinically defined MCI. Second, the intervention architecture was more consistent than theoretical labelling. Across studies, interventions commonly used education, goal setting, self-monitoring, feedback, prompts and cues, problem solving, reinforcement, and relational support. Third, theoretical fidelity was generally limited. Most interventions used components that were compatible with the TTM but did not measure stage progression, decisional balance, processes-of-change, or TTM-specific mediation. Fourth, outcome patterns were more favourable for proximal behavioural and implementation-related outcomes than for distal cognitive outcomes.

Clinically defined MCI was the anchor population for this review. The adjacent populations were informative because dementia-risk intervention research often occurs across a prevention continuum, before or around the emergence of measurable cognitive impairment. However, these groups should not be treated as interchangeable. Direct claims about TTM-based intervention effects in MCI are supported only by Shi et al. [17]. Findings from subjective cognitive concern, dementia-risk, midlife Alzheimer disease risk, and care-partner dyad studies are better interpreted as evidence about intervention architecture, feasibility, maintenance support, and outcome selection across the wider dementia-risk behaviour change literature.

The distinction between TTM-based, TTM-informed, and TTM-congruent studies is central to interpretation. Shi et al. [17] measured behavioural stage and delivered stage-aligned health education, allowing a direct but short-term assessment of TTM-based intervention effects. Gray Matters [15] was TTM-informed but did not test the full TTM mechanisms. Kim et al. [18], AIBL Active [19], AgeWell.de [21], MEDEX [20], Bryant et al. [23], and MedEx-UK [22] were TTM-congruent rather than TTM-based or TTM-informed. These studies share behaviour change components with the TTM, but most did not test the TTM’s mechanisms.

The behavioural findings are best interpreted across three levels. Engagement refers to participation, retention, app use, attendance, or acceptability. Maintenance refers to sustained adherence or continued participation over time. Actual behaviour change refers to a measurable change in the target behaviour. AIBL Active [19] provides strong evidence that long-term physical activity adherence can be achieved in selected cognitively at-risk older adults. Gray Matters [15] supports high digital engagement and behaviour monitoring. MedEx-UK [22] supports sustained Mediterranean diet adherence. AgeWell.de [21] supports improvement in selected surrogate dementia-risk components, particularly diet and hypertension. These findings are encouraging, but they do not establish that TTM-specific mechanisms caused the observed changes.

The cognitive findings are more limited. Shi et al. [17] reported improvement in global cognition by 20 weeks, and MedEx-UK [22] reported short-term improvements, in general, such as cognition and memory at 24 weeks. However, these findings must be interpreted alongside the Mindfulness, Education, and Exercise for Age-Related Cognitive Decline (MEDEX) trial [20], which was the largest included trial and found no significant improvement in episodic memory, executive function, or prespecified secondary outcomes at 6 or 18 months. Null findings do not exclude the possibility that behavioural interventions may require longer exposure, different intensity, better risk targeting, or more sensitive cognitive measures. They do, however, limit claims that structured behavioural interventions in cognitively at-risk populations reliably produce cognitive benefit.

This review also shows why the TTM remains clinically relevant but empirically under-tested. The TTM remains a useful focal model because it explicitly connects readiness, stage progression, self-efficacy, decisional balance, processes of change, relapse, and maintenance. These domains are central to dementia-risk behaviour change, where the challenge is not only knowing what risk factors to modify, but whether individuals are ready, confident, and supported enough to initiate and sustain change. However, common techniques such as goal setting, prompts, reminders, feedback, and social support are not unique to the TTM. They are also central to other behaviour change frameworks, including the Behaviour Change Wheel and behaviour change technique taxonomy. Future TTM studies in MCI should therefore measure the TTM constructs prospectively, test whether stage-matched support adds value beyond non-stage-matched behavioural support, and examine whether changes in behavioural stage, self-efficacy, or decisional balance mediate behavioural, functional, or cognitive outcomes.

### 4.1. Behavioural Mechanisms, Risk Modification, and the Cognitive-Risk Continuum

The pattern of findings in this review is best interpreted by distinguishing outcomes according to their proximity to intervention delivery. The most consistent findings were observed for proximal behavioural and implementation-related outcomes, including behavioural stage progression, disease knowledge, adherence, self-care, Mediterranean diet adherence, app engagement, sustained physical activity participation, and care-partner-supported daily routines. These outcomes sit closest to the recurrent intervention components identified across studies: education, goal setting, self-monitoring, prompts and cues, personalised feedback, problem solving, reinforcement, social support, and maintenance support.

This distribution of findings is clinically and methodologically coherent. Behavioural and implementation outcomes are more directly exposed to the intervention and are more likely to change within the short or intermediate follow-up periods used in the included studies. Cognitive outcomes are more distal. They are likely to depend on cumulative exposure, sustained adherence, baseline neuropathology, cognitive reserve, comparator intensity, intervention dose, and the sensitivity of outcome measures. The current evidence therefore supports a cautious interpretation: TTM-based and TTM-congruent interventions appear more consistently associated with behavioural regulation, engagement, and maintenance than with durable cognitive improvement.

Intermediate risk modification occupies a distinct position between behavioural change and cognitive outcomes. AIBL Active [19] supports the feasibility of sustained physical activity adherence over 24 months; AgeWell.de [21] demonstrated improvement in LIBRA dementia-risk scores, mainly through diet and hypertension; and MedEx-UK [22] showed sustained improvement in Mediterranean diet adherence at 24 and 48 weeks. These findings suggest that behaviourally structured interventions may support maintenance of health-related routines and selected dementia-risk markers even when cognitive effects are modest, inconsistent, or absent. This distinction is important because a reduction in modifiable risk exposure may be clinically meaningful in prevention and early cognitive vulnerability, but it should not be presented as equivalent to proven cognitive efficacy.

The applicability of these findings varies across the cognitive-risk continuum. Clinically defined MCI was the anchor population of this review. Studies involving subjective cognitive concerns, elevated dementia risk, midlife Alzheimer’s disease risk, or care-partner dyads were retained as adjacent evidence because dementia-prevention interventions are often delivered before, around, or shortly after measurable cognitive impairment emerges. These groups are not interchangeable. Interventions that are feasible in prevention-oriented or subjectively concerned adults may operate differently in established MCI, where memory impairment, executive dysfunction, reduced confidence, and reliance on caregivers may affect self-monitoring, goal tracking, and maintenance. Findings from adjacent cohorts, therefore, help map intervention architecture and theoretical fidelity, but direct claims about TTM-based effects in MCI should remain restricted to the clinically defined MCI evidence.

External scaffolding appeared central to sustained change. Across studies, behavioural effects were rarely attributable to information provision alone. More consistent behavioural signals occurred where interventions incorporated repeated cueing, structured routines, personalised feedback, problem solving, monitoring, relational support, or care-partner involvement. Bryant et al. [23] illustrate this most clearly: care-partner-mediated oral hygiene support relied on prompts and cues, instruction, review of behavioural goals, and problem solving. Gray Matters [15] similarly suggests that digital self-monitoring and personalised feedback can support engagement, although its motivational findings were subgroup-dependent. These findings are compatible with the TTM’s emphasis on readiness, self-efficacy, reinforcement, and maintenance, but they are not unique to the TTM. They also overlap with broader behaviour change frameworks and behaviour change technique taxonomies.

**Figure 2:**
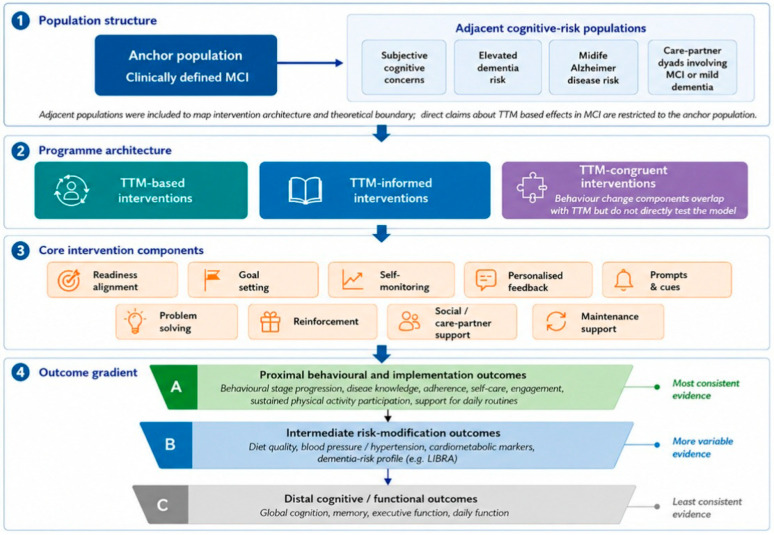
Evidence-derived framework of programme and outcome pathways.

Figure 2 should therefore be interpreted as an evidence-derived framework rather than a validated causal model. It summarises the observed gradient from intervention architecture to proximal behavioural outcomes, intermediate risk modification, and distal cognitive or functional outcomes. The evidence is strongest at the behavioural and implementation levels, more variable at the intermediate risk-modification level, and weakest for durable cognitive benefit.

### 4.2. Limitations

This review has several limitations. First, only one included study directly tested an explicit TTM-based intervention in clinically defined MCI. Most included studies were TTM-informed or TTM-congruent rather than TTM-based. Second, several intervention components mapped in this review, including goal setting, prompts, feedback, reinforcement, and social support, are not unique to the TTM and cannot be treated as evidence of TTM-specific mechanisms unless linked prospectively to TTM constructs. Third, although clinically defined MCI was treated as the anchor population, the evidence base also included adjacent cognitive-risk populations, including subjective cognitive concerns, elevated dementia risk, midlife Alzheimer disease risk, and care-partner dyads. These groups differ in cognitive profiles, autonomy, caregiver involvement, and likely response to intervention; therefore, the findings from adjacent groups cannot be assumed directly applicable to MCI. Fourth, many outcomes reflected engagement, adherence, acceptability, feasibility, or surrogate risk modification rather than objective sustained behaviour change or durable cognitive benefits. Fifth, formal risk-of-bias scoring was not undertaken, which is consistent with scoping review methodology, although structured appraisal was used to guide interpretation. Sixth, restriction to English-language publications may have excluded relevant studies.

### 4.3. Future Directions

Future studies should evaluate theories more explicitly. When the TTM is invoked, stages of change, self-efficacy, decisional balance, and processes of change should be measured longitudinally, and intervention components should be mapped prospectively to those constructs. This would allow clearer mechanistic inference and a stronger distinction between explicit TTM testing and broader behavioural structuring.

The field requires adequately powered trials in clinically defined MCI cohorts rather than continued reliance on mixed or predominantly at-risk samples. These studies should include longer follow-ups, prespecified cognitive and functional outcomes, and analytic models capable of testing whether behavioural changes and intermediate risk modifications are associated with later cognitive or functional outcomes. Recent subgroup analyses [24] in multidomain intervention research also indicate that response may vary by clinical and metabolic context, supporting prespecified assessments of effect modification in future trials.

Interventions should be designed in ways that are proportionate to cognitive vulnerability. The current evidence suggests that maintenance support, cueing, prompts, and caregiver involvement are important in this population. Future programmes should therefore incorporate simplified monitoring tools, structured routines, tailored feedback, and explicit care-partner integration where appropriate. This is particularly relevant for delivery in geriatric, primary care, and memory service settings. Recent scoping work on [25] coping and adaptation in MCI and mild dementia further supports the importance of reminders, contextual support, and adaptive routines.

Future studies should align outcomes more closely with clinically meaningful endpoints. Cognitive scores remain important, but they should be interpreted alongside behavioural adherence, daily function, treatment burden, caregiver burden, and the sustainability of behavioural change. In prevention-oriented populations, durable change in health-related routines may itself be clinically meaningful, even when short-term cognitive change is modest. Recent evidence [26] in subjective cognitive decline and multidomain prevention supports this broader outcome logic, with benefits sometimes clustering in selected domains rather than appearing uniformly across all cognitive measures.

The proposed framework should be examined prospectively in future studies. Trials should assess whether specific programme components are associated with proximal behavioural change, whether these changes predict intermediate risk modification, and whether changes at those levels are associated with later cognitive or functional outcomes. This would allow the framework to move from interpretive synthesis toward empirical testing.

## 5. Conclusions

The included studies support an evidence-derived framework that organises this literature across programme structure, recurrent behavioural components, proximal behavioural outcomes, intermediate health and risk effects, and distal cognitive outcomes. Within that framework, the most consistent findings lie in behavioural and intermediate domains rather than in the cognitive domain.

The current evidence indicates that TTM-based, TTM-informed, and TTM-congruent interventions are associated more consistently with improved knowledge, adherence, health-related behaviour, dietary changes, sustained engagement with physical activity programmes, and support for daily routines than with durable cognitive gain. Only a small proportion of the included evidence represents explicit TTM trials in clinically defined MCI. Most of the literature reflects structured behaviour change programmes that are compatible with TTM principles but do not formally test the TTM as the principal mechanism.

The available evidence supports the behavioural plausibility of these interventions for strengthening support, risk reduction, and maintenance of health-related routines in cognitively vulnerable populations. Evidence for durable cognitive efficacy remains limited and inconsistent. The proposed framework provides a structured basis for interpreting the present literature and for guiding future mechanistic and clinical studies rather than a validated causal model or a basis for TTM-specific clinical recommendations.

## Figures and Tables

**Table 1:** Characteristics of included studies and degree of TTM operationalisation.

Study	Population and Setting	Design and Sample	Intervention and Comparator	Actual Theoretical or Intervention Basis	TTM Category	TTM Constructs Implemented or Measured
Shi et al. 2025 [17]	Community-dwelling older adults with MCI in Huzhou, China	Assessor-blinded randomised controlled trial, *n* = 100	Weekly 45 to 60 min TTM-based health education for 8 weeks, followed by 12 weeks of unsupervised practice versus wait-list standard health education	TTM-based health education	TTM-based	Behavioural stage measured; stage-aligned education delivered; adherence and disease knowledge assessed; global cognition assessed. Decisional balance, temptation, and full processes-of-change mediation were not reported as separate construct-level tests.
Kim et al. 2019 [18]	Older adults with MCI in the community; South Korea	Quasi-experimental nonequivalent control group pre–post design	Self-care intervention programme versus comparison condition	Simmons’s Health-Promoting Self-Care System Model, informed by Orem, Pender, and Cox	TTM-congruent	No TTM stages measured. Self-efficacy, dementia-preventive behaviour, cognition, depression, and QoL were assessed.
Cox et al. 2019 [19]	Older adults with MCI or subjective memory complaints and at least one cerebrovascular risk factor; Australia	Randomised controlled trial, *n* = 106	24-month home-based physical activity programme versus usual lifestyle control	Home-based physical activity behavioural support	TTM-congruen	No TTM constructs measured. Adherence, retention, PA, fitness, and body-composition outcomes assessed.
Hartin et al. [15]. 2016, Gray Matters primary report	Midlife adults at elevated Alzheimer’s disease risk; United States	Randomised controlled trial, treatment *n* = 102, control *n* = 42	Smartphone app-based multidomain behavioural intervention versus control	Digital education, self-monitoring, feedback, and reinforcement; app design includes TTM-aligned reinforcement language	TTM-informed	Self-monitoring, recommended goals, feedback, and reinforcement were used. No full TTM stage progression or construct-level mediation was reported.
Schiwal et al. [16]. 2020, Gray Matters secondary analysis	Same Gray Matters trial cohort with complete motivation data	Secondary analysis of the Gray Matters randomised trial	Same intervention, analysed with emphasis on motivation and behavioural change	Self-determination theory motivational analysis of the Gray Matters parent trial	TTM-informed	Intrinsic motivation, amotivation, external regulation, and identified regulation were assessed. No TTM stage testing.
Zülke et al. 2024 [21]	Older adults at increased dementia risk; Germany	Secondary analysis of the AgeWell.de trial, *n* = 461, with complete 24-month LIBRA data	Multicomponent intervention, including optimisation of nutrition, medication, and physical, social, and cognitive activity	Multidomain dementia-risk intervention	TTM-congruent	No TTM constructs measured. LIBRA risk score and individual risk components assessed.
Lenze et al. 2022 [20]	Community-dwelling adults aged 65 to 84 years with subjective cognitive concerns and no dementia, MCI permitted; United States	Two by two factorial randomised clinical trial, *n* = 585	Mindfulness-based stress reduction, exercise, combined intervention, or health education control over 18 months	Mindfulness, exercise, and health education interventions	TTM-congruent	No TTM constructs measured. Attendance/completion and cognitive outcomes assessed.
Bryant et al. 2024 [23]	Seventeen care-partner dyads involving persons with MCI or mild dementia; United States	Qualitative study using directed and emergent coding of coaching transcripts	Oral hygiene intervention delivered through nurse-led coaching with care partners	Behaviour change technique-based, TTM-congruent	TTM-congruent	Prompts/cues, instruction, behavioural goal review, and problem solving were identified. No TTM stage testing.
Jennings et al. 2024 [22]	Older adults at risk of dementia; United Kingdom	Three-arm randomised controlled trial, *n* = 104	Personalised theory-informed intervention targeting the Mediterranean diet alone or Mediterranean diet plus physical activity, with optional follow-ups	Personalised diet/physical activity intervention using web-based support, group sessions, food provision, and optional maintenance follow-up	TTM-congruent	Personalised targets, behavioural support, and maintenance follow-up were used. No TTM constructs measured.

**Table 2:** Behavioural, cognitive, and related findings of included studies.

Study	Behavioural or Engagement-Related Findings	Cognitive Findings	Other Relevant Findings and Interpretive Considerations
Shi et al. 2025 [17]	Behavioural stage improved at 8 weeks and 20 weeks: β8w = 1.04, 95% CI 0.34–1.75; β20w = 1.72, 95% CI 0.95–2.49. Disease knowledge improved: β8w = 1.14, 95% CI 0.26–2.02; β20w = 1.78, 95% CI 0.87–2.69. Adherence improved: β8w = 6.20, 95% CI 2.03–10.37; β20w = 10.74, 95% CI 6.47–15.01.	Global cognition improved significantly at 20 weeks: β20w = 2.42, 95% CI 1.64–3.20. The 8-week estimate crossed the null: β8w = 0.60, 95% CI −0.18 to 1.38. Purdue Pegboard Assembly and Bimanual Tasks improved at 20 weeks. Depression and sleep quality did not show significant individual-level improvement.	Only direct TTM-based MCI trial. Supports short-term behavioural and cognitive behavioural plausibility, not durable cognitive efficacy.
Kim et al. 2019 [18]	Significant group-by-time effects for dementia-preventive behaviour: F = 103.28, *p* < 0.001 from baseline to week 5; F = 18.31, *p* = 0.040 from week 5 to week 9. Self-efficacy: F = 86.91, *p* < 0.001 from baseline to week 5; F = 7.34, *p* = 0.009 from week 5 to week 9.	Cognition improved from baseline to week 5, F = 23.05, *p* < 0.001, but not from week 5 to week 9, F = 0.15, *p* = 0.692. Depression improved from baseline to week 5, F = 28.29, *p* < 0.001, but not from week 5 to week 9, F = 1.04, *p* = 0.312. QoL improved from baseline to week 5, F = 48.47, *p* < 0.001, but not from week 5 to week 9, F = 0.93, *p* = 0.338.	TTM-congruent self-care study, not TTM-based. Quasi-experimental design, self-report, and lack of blinding limit causal inference.
Cox et al. 2019 [19]	24-month retention was 97.2%. Median prescribed PA adherence was 91.67% (Q1–Q3 81.96–100.00), with no significant variation over time (*p* = 0.90). Median total PA adherence was 81.83% (Q1–Q3 69.38–92.29), with no significant variation over time (*p* = 0.30).	Cognitive outcomes were not the focus of this report.	Strong evidence for adherence feasibility and behavioural maintenance in selected at-risk older adults; not a TTM test.
Hartin et al. [15]. 2016, Gray Matters primary report	Mean app use was 7.3 behavioural logs/day. HDL improvers answered more questions/day than non-improvers: 8.30 vs. 6.52; t97.74 = −3.051, *p* = 0.003. BMI improvers achieved a higher proportion of recommended daily goals than BMI non-improvers: 56.21% vs. 40.12%; t80 = −2.449, *p* = 0.017.	No direct cognitive efficacy outcome.	Supports digital engagement and behaviour monitoring; does not establish TTM or cognitive efficacy.
Schiwal et al. [16]. 2020, Gray Matters secondary analysis	Intrinsic motivation increased more in treatment than control: 2.09 vs. 1.00; t130 = −3.04, *p* = 0.003. High intrinsic motivation males increased vigorous PA more than lower intrinsic motivation males: F1,42 = 5.053, *p* = 0.030. Older high intrinsic motivation participants had less DASH diet improvement: F1,48 = 4.538, *p* = 0.038.	No cognitive efficacy outcome.	Motivational signal was mixed and subgroup-dependent. Supports caution against a simple motivation-to-behaviour pathway.
Zülke et al. 2024 [21]	LIBRA score improved: b = −0.63, 95% CI −1.14 to −0.12. Effects were mainly attributable to diet: OR 1.60, 95% CI 1.16–2.22; and hypertension: OR 1.61, 95% CI 1.19–2.18.	Surrogate dementia-risk outcome; not direct cognitive efficacy.	Supports selected risk-factor modification, not TTM-specific efficacy
Lenze et al. 2022 [20]	Trial completion was 97.1% at 6 months and 81.2% at 18 months.	No significant effects at 6 months on episodic memory: MBSR difference −0.04, 95% CI −0.15 to 0.07, *p* = 0.50; exercise difference 0.07, 95% CI −0.04 to 0.17, *p* = 0.23. No significant effects on executive function: MBSR difference 0.08, 95% CI −0.02 to 0.19, *p* = 0.12; exercise difference 0.07, 95% CI −0.03 to 0.18, *p* = 0.17. No intervention effects were observed at 18 months, and none of the five prespecified secondary outcomes improved significantly.	Largest included trial. Key null finding limiting claims about cognitive benefit.
Bryant et al. 2024 [23]	In 17 dyads, the most frequent BCTs were prompts and cues, instructions on how to perform the behaviour, review of behavioural goals, and problem solving. Social support emerged in the MCI group; credible source emerged in the mild dementia group.	No cognitive outcomes were assessed	Clarifies care-partner-mediated behavioural mechanisms; no TTM construct testing.
Jennings et al. 2024 [22]	Mediterranean diet adherence improved by 3.7 points on a 14-point scale at 24 weeks and 2.7 points at 48 weeks compared with the control. Objectively measured PA did not significantly change.	General cognition improved at 24 weeks: 0.22, 95% CI 0.05–0.35; memory improved: 0.31, 95% CI 0.10–0.51. Cognitive differences were not sustained at 48 weeks.	Supports domain-specific dietary behaviour changes more strongly than PA change or durable cognitive benefit.

## Data Availability

No new data were created or analyzed in this study.

## Supplementary Materials

The following supporting information can be downloaded at: https://www.mdpi.com/article/10.3390/healthcare14131898/s1.
