# Supplementary material for: Transtheoretical Model (TTM)-Based, TTM-Informed and TTM-Congruent Behaviour Change Interventions for Adults with Mild Cognitive Impairment and Dementia Risk: A Scoping Review
Source: Healthcare (Basel). 2026 Jun 30;14(13):1898. doi: 10.3390/healthcare14131898 (PMC13362339; doi:10.3390/healthcare14131898)
Supplement: Supplementary file 1 [file healthcare-14-01898-s001.zip › Supplementary Index S1.pdf]

Supplementary Index S1. PubMed search strategy

Database: PubMed
Platform: PubMed, National Library of Medicine/National Center for Biotechnology Information
Date searched: 30 September 2025
Coverage: Inception to 30 September 2025
Limits applied: Humans; English language; date filter as applied in PubMed
Records retrieved: 1456
Note: Line breaks and indentation are added for readability. Search terms, field tags, Boolean operators, and limits reflect the final PubMed search strategy.

Full PubMed search strategy

```
(
  "mild cognitive impairment"[tiab] OR
  MCI[tiab] OR
  "amnestic MCI"[tiab] OR
  "cognitive decline"[tiab] OR
  "mild neurocognitive disorder"[tiab] OR
  "prodromal dementia"[tiab] OR
  "cognitive impairment not dementia"[tiab] OR
  CIND[tiab] OR
  "borderline cognitive function"[tiab] OR
  "Mini-Mental State Examination"[tiab] OR
  MMSE[tiab] OR
  "Montreal Cognitive Assessment"[tiab] OR
  MoCA[tiab] OR
  "Global Deterioration Scale"[tiab] OR
  GDS[tiab] OR
  "Clinical Dementia Rating"[tiab] OR
  CDR[tiab] OR
  "CDR = 0.5"[tiab] OR
  MoCA[tiab] AND score[tiab] AND (less[tiab] OR below[tiab]) AND 26[tiab] OR
  MMSE[tiab] AND score[tiab] AND (greater[tiab] OR above[tiab]) AND 24[tiab] OR
  "trail making test"[tiab] OR
  "category fluency"[tiab] OR
  "delayed recall"[tiab] OR
  "immediate recall"[tiab] OR
  (standard deviation[tiab] AND below[tiab] AND normal[tiab]) OR
```

```
  "Cognitive Dysfunction"[MeSH] OR
  "Neurocognitive Disorders"[MeSH]
)
AND
(
  "transtheoretical model"[tiab] OR
  TTM[tiab] OR
  "stages of change"[tiab] OR
  "stage-matched intervention"[tiab] OR
  "readiness to change"[tiab] OR
  "health behaviour change"[tiab] OR
  "precontemplation"[tiab] OR
  "contemplation"[tiab] OR
  "preparation"[tiab] OR
  "action"[tiab] OR
  "maintenance"[tiab] OR
  "termination"[tiab] OR
  "consciousness raising"[tiab] OR
  "self-reevaluation"[tiab] OR
  "environmental reevaluation"[tiab] OR
  "self-liberation"[tiab] OR
  "social liberation"[tiab] OR
  "helping relationships"[tiab] OR
  "counterconditioning"[tiab] OR
  "reinforcement management"[tiab] OR
  "stimulus control"[tiab] OR
  "dramatic relief"[tiab] OR
  "decisional balance"[tiab] OR
  "self-efficacy"[tiab] OR
  "temptation"[tiab] OR
  "Transtheoretical Model"[MeSH] OR
  "Health Behavior"[MeSH] OR
  "Health Education"[MeSH] OR
  "Patient Education as Topic"[MeSH]
)
AND
(
  "cognitive function"[tiab] OR
```

"executive function"[tiab] OR
"working memory"[tiab] OR
"processing speed"[tiab] OR
"episodic memory"[tiab] OR
"verbal fluency"[tiab] OR
"attention"[tiab] OR
"mental health"[tiab] OR
"psychological wellbeing"[tiab] OR
"emotional wellbeing"[tiab] OR
"mood"[tiab] OR
"anxiety"[tiab] OR
"depression"[tiab] OR
"self-esteem"[tiab] OR
"activities of daily living"[tiab] OR
"instrumental activities of daily living"[tiab] OR
"functional independence"[tiab] OR
"functional capacity"[tiab] OR
"gait speed"[tiab] OR
"timed up and go"[tiab] OR
"dual tasking"[tiab] OR
"balance test"[tiab] OR
SPPB[tiab] OR
"chair rise"[tiab] OR
"motor function"[tiab] OR
"treatment adherence"[tiab] OR
"intervention adherence"[tiab] OR
"health behaviour"[tiab] OR
"engagement"[tiab] OR
"motivation"[tiab] OR
Neuropsychiatric Inventory[tiab] OR
"caregiver observation"[tiab] OR
"informant report"[tiab] OR
"caregiver questionnaire"[tiab] OR
"quality of life"[tiab] OR
QOL[tiab] OR
"life satisfaction"[tiab] OR
"health-related quality of life"[tiab] OR
HRQoL[tiab] OR

wellbeing[tiab] OR
"EQ-5D"[tiab] OR
"EQ-5D-5L"[tiab] OR
"EQ5D3L"[tiab] OR
EuroQol[tiab] OR
WHOQOL[tiab] OR
"WHOQOL-BREF"[tiab] OR
"QOL-AD"[tiab] OR
"SF-36"[tiab] OR
"SF-12"[tiab] OR
"Health Utilities Index"[tiab] OR
HUI[tiab] OR
"Cognition"[MeSH] OR
"Memory"[MeSH] OR
"Executive Function"[MeSH] OR
"Activities of Daily Living"[MeSH] OR
"Mental Health"[MeSH] OR
"Treatment Adherence and Compliance"[MeSH] OR
"Depression"[MeSH] OR
"Anxiety"[MeSH] OR
"Gait"[MeSH] OR
"Postural Balance"[MeSH] OR
"Quality of Life"[MeSH] OR
"Health Status Indicators"[MeSH] OR
"Caregivers/psychology"[MeSH]
)
AND
(
memory[tiab] AND clinic[tiab] OR
cognitive[tiab] AND (clinic[tiab] OR centre[tiab]) AND (rehabilitation[tiab] OR intervention[tiab] OR training[tiab]) OR
pharmacological[tiab] AND (treatment[tiab] OR intervention[tiab]) AND MCI[tiab] OR
psychoeducation[tiab] OR
cognitive[tiab] AND stimulation[tiab] OR
"standard care"[tiab] OR
"usual care"[tiab] OR
"routine care"[tiab] OR
"care as usual"[tiab] OR

non-TTM[tiab] OR
(non[tiab] AND behavioural[tiab] AND intervention[tiab]) OR
(non[tiab] AND TTM[tiab] AND program[tiab]) OR
"waitlist"[tiab] OR
"no treatment"[tiab] OR
"placebo"[tiab] OR
"attention control"[tiab] OR
"control group"[tiab] OR
"comparison group"[tiab] OR
"inactive comparator"[tiab] OR
"Treatment Outcome"[MeSH] OR
"Memory Disorders/therapy"[MeSH] OR
"Ambulatory Care"[MeSH] OR
"Outpatient Clinics, Hospital"[MeSH] OR
"Placebos"[MeSH] OR
"Control Groups"[MeSH]
)
