# Supplementary material for: Transtheoretical Model (TTM)-Based, TTM-Informed and TTM-Congruent Behaviour Change Interventions for Adults with Mild Cognitive Impairment and Dementia Risk: A Scoping Review
Source: Healthcare (Basel). 2026 Jun 30;14(13):1898. doi: 10.3390/healthcare14131898 (PMC13362339; doi:10.3390/healthcare14131898)
Supplement: Supplementary file 1 [file healthcare-14-01898-s001.zip › Supplementary Index S2.pdf]

Study	Strengths	Main limitations for interpretation
Shi et al. 2025	Assessor-blinded RCT; clinically defined MCI; explicit TTM operationalisation; construct-relevant outcomes.	Short follow-up; wait-list control; limited construct-level mediation; durability uncertain.
Kim et al. 2019	Clinically defined MCI population; self-care intervention; multiple behavioural and psychosocial outcomes.	Nonequivalent control-group quasi-experimental design; small sample; self-report; no blinding; not TTM-based.
Cox et al. 2019	Randomised design; 24-month follow-up; strong adherence and retention data.	Secondary outcome report; selected motivated cohort; not TTM-based; limited TTM construct relevance.
Hartin et al. 2016	RCT; high-resolution app engagement data; objective cardiometabolic markers.	Midlife prevention cohort without significant cognitive impairment; indirect behavioural associations; no cognitive efficacy endpoint.
Schiwal et al. 2020	Randomised parent trial; formal motivation measures.	Secondary analysis; subgroup findings; SDT rather than TTM; no cognitive endpoint.
Zülke et al. 2024	Large pragmatic multidomain trial; 24-month follow-up; dementia-risk score outcome.	Secondary analysis; surrogate risk outcome; not TTM-based; incomplete improvement across risk domains.
Lenze et al. 2022	Largest included RCT; factorial design; long follow-up; prespecified cognitive outcomes.	Subjective cognitive concerns rather than clinically defined MCI; not TTM-based; intervention may not have targeted dementia-risk mechanisms sufficiently.
Bryant et al. 2024	Qualitative mechanism-focused study; dyads with MCI and mild dementia; BCT coding.	Small sample; no cognitive outcomes; no TTM construct testing; care-partner-mediated context limits generalisability.
Jennings et al. 2024	RCT; personalised diet/PA intervention; behaviour-	Dementia-risk rather than MCI cohort; small sample; PA did not change

Study	Strengths	Main limitations for interpretation
	maintenance follow-up; diet and cognition outcomes.	objectively; cognitive effects not sustained at 48 weeks.
